# Development of a sensitive RT-PCR method for amplifying and sequencing near full-length HCV genotype 1 RNA from patient samples

**DOI:** 10.1186/1743-422X-10-53

**Published:** 2013-02-12

**Authors:** Eileen Z Zhang, Doug J Bartels, J Dan Frantz, Sheila Seepersaud, Judith A Lippke, Benjamin Shames, Yi Zhou, Chao Lin, Ann Kwong, Tara L Kieffer

**Affiliations:** 1 Vertex Pharmaceuticals Incorporated, 130 Waverly Street, Cambridge, MA, 02139-4242, USA

**Keywords:** Hepatitis C Virus (HCV), Reverse Transcription Polymerase Chain Reaction (RT-PCR), Genotype, Direct Acting Antiviral Agents (DAA), Lower Limit of Detection (LLOD), Quasispecies, Sequencing, Resistance, NS (Non-structural), NS3/4A, NS4B, NS5A, NS5B

## Abstract

**Background:**

Direct-acting antiviral (DAAs) agents for hepatitis C virus (HCV) span a variety of targets, including proteins encoded by the NS3/4A, NS4B, NS5A, and NS5B genes. Treatment with DAAs has been shown to select variants with sequence changes in the HCV genome encoding amino acids that may confer resistance to the treatment. In order to assess these effects in patients, a Reverse Transcription Polymerase Chain Reaction (RT-PCR) method was developed to sequence these regions of HCV from patient plasma.

**Methods:**

A method was developed to amplify and sequence genotype 1 HCV RNA from patient plasma. Optimization of HCV RNA isolation, cDNA synthesis, and nested PCR steps were performed. The optimization of HCV RNA isolation, design of RT-PCR primers, optimization of RT-PCR amplification conditions and reagents, and the evaluation of the RT-PCR method performance is described.

**Results:**

The optimized method is able to successfully, accurately, and reproducibly amplify near full-length genotype 1 HCV RNA containing a wide range of concentrations (10^3^ to 10^8^ IU/mL) with a success rate of 97%. The lower limit of detection was determined to be 1000 IU/mL HCV RNA.

**Conclusions:**

This assay allows viral sequencing of all regions targeted by the most common DAAs currently in development, as well as the possibility to determine linkage between variants conferring resistance to multiple DAAs used in combination therapy.

## Background

More than 170 million people (3% of the world population) are infected with hepatitis C virus (HCV) [1]. Chronic HCV infection is a major cause of liver cirrhosis and hepatocellular carcinoma (HCC), as well as liver failure. Liver failure is one of the most common indications for liver transplantation in the United States [2,3]. The recent development of potent, direct-acting antiviral agents (DAAs), used in combination with pegylated-interferon (Peg-IFN) and ribavirin (RBV) has significantly improved the sustained virologic response rates compared with Peg-IFN and RBV alone [4,5].

HCV has high genetic variability across the six major genotypes as well as in different subtypes within each genotype. Genotype 1, the predominant cause of chronic HCV infections in the United States, Europe, and Japan, has more diversity within subtypes 1a and 1b than the entire diversity of HBV and HIV [6]. In an HCV-infected patient, this pre-existing genetic variability provides a pool of variants that can adapt to new selection pressures, such as antiviral treatment. The variants are a natural part of the viral quasispecies, which arise from the error-prone nature of the HCV RNA-dependent RNA polymerase and the high replication rate of the virus [7]. In patients who fail treatment with a potent DAA regimen, the elimination of the majority of the DAA-sensitive quasispecies can lead to selection and detection of DAA-resistant variants [8].

The nonstructural (NS) HCV proteins NS3, NS4A, NS4B, NS5A, and NS5B are essential for viral replication and are the major targets of DAAs currently in development [9]. Thus, monitoring viral sequence changes across multiple NS regions is beneficial in understanding the potential association between therapeutic resistance and clinical outcome [10].

Various methods for amplification and sequence analysis of specific HCV genomes have been reported, and most of them have focused on individual NS segments or smaller amplicons [11-14]. In 2006, a long reverse transcription polymerase chain reaction (RT-PCR) amplification protocol of a near full-length HCV genome was developed by Fan, X, Xu, Y, and Di Bisceglie, AM, that amplified a limited number of patient samples [15-17]. While conducting telaprevir (INCIVEK™, INCIVO®) clinical trials [18,19], the need arose to develop a method to analyze the HCV genotype 1 sequence of NS3, NS4A,NS5A, and NS5B from a single genome or the catalytic domain of the NS3·4A protease domain from a large number of patients. The challenge was that samples from patients are extremely variable and existing methods described in the literature required patient-by-patient optimization to efficiently amplify near full-length HCV RNA, particularly in samples with low HCV RNA levels, which were common in patients who were responding to telaprevir and/or VX-222. In this study, we describe the development of an efficient RT-PCR method that was used in telaprevir clinical trials to amplify a nearly full-length HCV genotype 1 genome from 7800 unique patient samples containing a wide range of HCV RNA concentrations (10^3^ to 10^8^ IU/mL) with a success rate of 97% [4,5,20-23]. This method enables HCV genes, targeted by the most common DAAs in clinical trials today, to be efficiently sequenced using clinical samples with low HCV RNA levels and permits the assessment of linkage between multiple amino acid changes in different HCV genes in samples from patients receiving DAA drug combinations.

## Results and discussion

### Optimization of HCV RNA extraction

The use of the 96-well format QIAamp Virus BioRobot 9604 Kit (QIAGEN, Valencia, CA) was optimized to produce high-quality viral RNA from HCV RNA-positive plasma. Several modifications were made, with the primary change involving the replacement of the carrier RNA in the RNA isolation. Comparison of the use of poly A RNA (rA) provided in the QIAGEN kit with transfer RNA (tRNA) (Roche Applied Science, Indianapolis, IN) or ribosomal RNA (rRNA) (Roche Applied Science, Indianapolis, IN) using plasma samples with a wide range of HCV RNA copy numbers (10 ~ 18,500 HCV RNA copies/reaction) showed that 60 μg of tRNA per column for 660 μL plasma led to the highest level of sensitivity in the subsequent amplification reaction. In addition, several other minor changes to the standard QIAamp protocol also increased viral RNA purity and quality, which helped the performance of subsequent amplifications including mixing via gentle rocking, instead of pipetting (per the BioRobot 9604 Kit protocol), to minimize shearing of the 9.6 kb HCV RNA. Also, for optimal recovery of viral RNA, the samples were lysed at 56°C and an additional spin step was added following the final wash step to eliminate residual ethanol carry-over.

### cDNA synthesis

Synthesis of cDNA is a critical step in the ability to amplify large PCR products, such as the 9.6 kb HCV genome. Optimization of the cDNA synthesis from HCV RNA involved testing different oligonucleotide primers including random hexamers, oligo d(A) (20 nucleotide), and primers that annealed in the NS5B or 3’ untranslated region (UTR). Surprisingly, most of the primers binding in the NS5B or 3’UTR did not allow amplification of full-length viral RNA or lead to very low cDNA yields. In contrast, oligo dA20, which anneals to the highly conserved poly (U) tail at the 3’ end of the HCV RNA, did allow for high sensitivity and reproducible amplification of full-length cDNA. We also tested several conditions and concentrations of Reverse Transcriptase enzyme (Superscript™ III Reverse Transcriptase [Life Technologies Corporation, Carlsbad, CA]) and it was determined that the use of 400 units with a 2.5 hour protocol, containing increasing annealing/elongation temperatures, was preferred.

### Amplification of HCV via nested PCR

**Figure 1:**
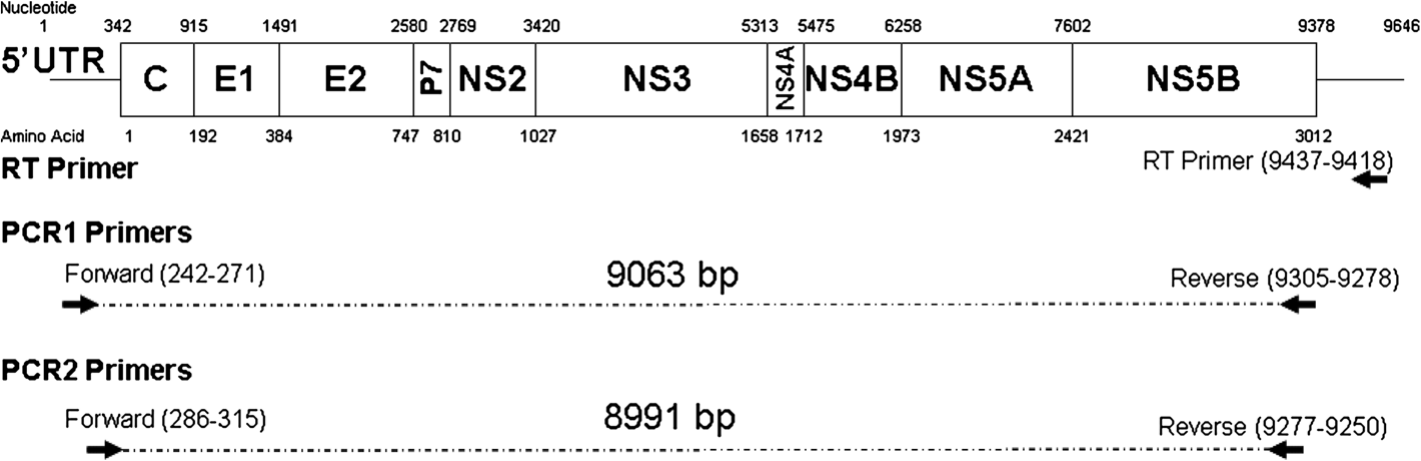
**Primer locations.** RT, PCR1 and PCR2 primers were designed based on H77 for subtype 1a and Con1 for subtype 1b, and the relative position of these primers is shown.

To amplify sufficient amounts of the HCV genome for subsequent sequence analysis, PCR primers and conditions were optimized. Using several hundred publicly available genotype 1 HCV sequences (Los Alamos HCV Sequence Database [24]), the regions of highest conservation in the 5’ and 3’UTRs were selected for potential primer annealing sites. Subsequent evaluation of primers to the conserved regions showed a diversity of responses in the assay. Many primers resulted in either a poor PCR yield, potentially due to the secondary structure of HCV RNA in the binding region, or a poorer than expected effectiveness across a spectrum of multiple samples. The highest performing primer annealing sites were at base pair (bp) 242–271 in the 5’UTR and bp 9278–9305 in the NS5B coding region. When various primer lengths were investigated, the use of 28–30 bp oligonucleotide primers led to the highest sensitivity and amplification across a spectrum of varied samples, by potentially annealing to viral genomes with several nucleotide mismatches from the primer. Also, to increase the ability to amplify across a spectrum of samples from a large number of patients, some degeneracy was added in the least conserved nucleotides in each annealing region. Differences in the primer annealing regions between subtypes 1a and 1b led to the design of subtype-specific primers (Table 1). Conditions with the largest impact on assay performance included the use of a touchdown PCR method (ramping annealing temperatures) for increased specificity, and addition of a second nested PCR reaction to increase the sensitivity of the assay to ~1000 IU/mL of HCV RNA (Figure 1). The combination of a highly processive polymerase (KlenTaq1™) with a proofreading (*pfu*) polymerase, and addition of 1.5 M betaine increased the yield of the ~9000 bp PCR product. Betaine is likely acting through the reduction of the bp composition dependence on DNA strand melting [25,26].

**Table 1:** List of primers used for the amplification of the HCV coding region

RT primer		Oligo d(A)	5’-AAAAAAAAAAAAAAAAAAAA-3’
PCR1 primers	Subtype 1a	GEN1.F1:	5’-CAAGACTGCTAGCCGAGTAGTGTTGGGTCG-3’
		GEN1A.R1	5’-CCGGGCAYGAGACACGCTGTGATAAATG-3’
	Subtype 1b	GEN1.F1	5’-CAAGACTGCTAGCCGAGTAGTGTTGGGTCG-3’
		GEN1B.R1	5’-TCGGGCACGAGACAVGCTGTGATATATG-3’
PCR2 primers	Subtype 1a	GEN1.F2	5’-GTACTGCCTGATAGGGTGCTTGCGAGTGCC-3’
		GEN1A.R2	5’-TCTCCCCCGCTGTAGCCAGCCGTGAACC-3’
	Subtype 1b	GEN1.F2	5’-GTACTGCCTGATAGGGTGCTTGCGAGTGCC-3’
		GEN1B.R2	5’-TCTCCCCCGCTGTARCCAGCRACGAACC-3’

### Sensitivity of RT-PCR amplification

To assess the sensitivity of the method, four genotype 1 HCV RNA-positive plasma samples (two subtypes 1a and two subtypes 1b) were serially diluted with concentrations of HCV RNA ranging from 11–649,000 IU/mL, and two analysts performed the amplification on different days. The HCV RNA-positive plasma samples were processed through the RT-PCR step and subsequent PCR amplifications were performed in triplicate. Samples with HCV RNA greater than or equal to 1000 IU/mL had an amplification success rate of 70% (100 out of 143 samples) in the first attempt. Samples with HCV RNA below 1000 IU/mL had a much lower amplification success rate of 1.6% (2 out of 121 samples). Therefore, the lower limit of detection (LLOD) of the RT-PCR amplification method was established to be ~1000 IU/mL. No significant differences in success rates were observed either between subtype 1a and 1b samples or between analysts.

### Robustness and success rate of amplification and sequencing

The performance of the assay was subsequently evaluated on a large, diverse set of plasma samples containing HCV subtypes la or lb from telaprevir and VX-222 clinical trials, with HCV RNA titers ranging from 100 IU/mL to greater than 10^7^ IU/mL (Table 2). We processed 7800 unique patient plasma samples containing subtype 1a (5400) and subtype 1b (2400) with HCV RNA at or above the assay limit of detection of 1000 IU/mL from August 2006 to December 2010. The genetic diversity of both subtypes 1a and 1b sample sets was similar to sample sets in the public domain (Los Alamos HCV Sequence Database [24]) as shown in Table 3 (Shannon Entropy [27], BioEdit version 7.0.5.3). The majority (~97%) of subtype 1a and subtype 1b samples were successfully amplified, and usually on the first attempt. For the remaining 3% of samples that failed to amplify, no correlation with HCV RNA level was observed. The failure of the assay was likely due to high sequence diversity in one or more of the primer binding regions, caused by either the natural high variability in genotype 1 samples, or mis-genotyping of the samples. Testing of samples below the 1000 IU/mL assay limit of detection, including samples as low as 100 IU/mL, led to a lower success rate (48% and 40% for subtypes 1a and 1b, respectively), and were not pursued further. Among the amplified samples, nearly 99% of the samples were successfully sequenced with a small set of standardized sequencing oligonucleotides, and the sequence information was consistent across replicates. The remaining 1% of samples for which sequence was not available had the highest sequence diversity in the areas of interest, leading to poor annealing for the sequencing oligonucleotides.

**Table 2:** Summary of RT-PCR amplification and sequencing success rate of clinical samples from telaprevir and VX-222 clinical trials

**HCV RNA (IU/mL)**^**a**^	**Genotype/Subtype**^**b**^	**Numbers of clinical samples**	**Success rate of RT-PCR amplification % (n/N)**	**Success rate of sequencing % (n/N)**
≥ 1000	1a	5400	97 (5238/5400)	99.6 (5218/5238)
	1b	2400	97 (2328/2400)	99.6 (2318/2328)
100–1000	1a	152	48 (73/152)	98.6 (73/73)
	1b	107	40 (43/107)	100.0 (43/43)

^a^HCV RNA was assessed using the Roche TaqMan HCV RNA assay (Version 2.0).

^b^Genotype and subtype was defined using 5'NC assay (Trugene) or Versant HCV genotype assay (LiPA) 2.0.

**Table 3:** Comparison of HCV NS3(4A) region of Los Alamos and vertex sequences

**Subtype**	**Sequence domain**	**Number of sequences°**	**Length of alignment (base pair)**	**Mean Shannon entropy**	**Average pairwise difference (Standard deviation)**
1a	Los Alamos Sequences	695	2067	0.132014	7.5% (± 1.9%)
	Vertex Sequences	2111*	2085	0.175243	9.1% (± 4.6%)
1b	Los Alamos Sequences	561	2067	0.158275	9.3% (± 1.5%)
	Vertex Sequences	1335†	2085	0.202466	10.6% (± 3.4%)

° Sequences of HCV NS3(4A) region.

* GenBank accession numbers: KC125008-KC127118.

† GenBank accession numbers: KC123434-KC124768.

## Conclusion

Despite the high sequence diversity of HCV, successful amplification of a near full-length (~9 kb) HCV genotype 1 genome was completed on 97% (7566/7800) of patient plasma samples with HCV RNA concentrations greater than or equal to 1000 IU/mL. This method facilitated the examination of sequence changes in the viral population during DAA treatment and aided the development of telaprevir [28]. The ability to amplify and sequence the near full length HCV genome and to look at mutations across multiple DAA targets will be important in the development of multiple DAA-based regimens.

## Methods

Studies included in these analyses were conducted in full compliance with the guidelines of Good Clinical Practice and of the World Medical Assembly Declaration of Helsinki. Prior to study initiation, protocols and informed consent forms were reviewed and approved by institutional review boards at each study site. All patients provided written informed consent before participating in any study-related activity. ClinicalTrials Identifiers for studies included in the manuscript are as follows: NCT00336479, NCT00372385, NCT00420784, NCT00535847, NCT00627926, NCT00983853, NCT00758043, NCT00916474, NCT00528528, NCT00703118, NCT00911963, NCT01080222.

This method is based on the amplification protocol originally described in Kwong et al. [29].

### HCV RNA extraction

Viral RNA was extracted from plasma in a 96-well format according to the manufacturer’s instruction with several modifications. Plasma (220 ~ 660 μL, the volume was adjusted based on viral load, i.e., 220 μL for HCV RNA greater than or equal to 50,000 IU/mL, while 660 μL for HCV RNA less than 50,000 IU/mL) was mixed with QIAGEN Protease K (40 ~ 120 μL) and QIAamp AL buffer (240 ~ 720 μL) supplemented with 20 ~ 60 μg of carrier RNA (tRNA) per column. The mixture was incubated for 15 min at 56°C, and absolute ethanol (293 ~ 875 μL) was added to each well and mixed well. The mixture was loaded into QIAamp column and passed through the column by suction with a peristaltic micropump (IPS-16; Ismatec, Zurich, Switzerland). Subsequently, the column was washed with AW1 buffer (1000 μL) and AW2 buffer (1000 μL) (provided in QIAGEN kit), respectively, by applying vacuum. A second wash with AW2 buffer (1000 μL) was spun at 6000 × g for 10 min and an additional spin at 6000 × g for 15 min was applied to remove residual ethanol. 40 μL of RNA storage solution (Ambion) was loaded into each column and incubated at room temperature for 5 min. The RNA was eluted by centrifugation at 6000 × g for 10 min. The elution was repeated once to increase the yield. A total of 80 μL viral RNA was isolated from 220 to 660 μL of plasma.

### cDNA synthesis

A complementary DNA (cDNA) fragment was synthesized from HCV RNA that was diluted (1:2 or 1:4, the dilution factor was based on viral load, i.e., 1:2 and 1:4 for HCV RNA less than 50,000 IU/mL, while 1:4 for HCV RNA greater than or equal to 50,000 IU/mL) into a total RT reaction volume of 20 μL composed of 2.5 μM of an oligo-dA20 primer (Table 1), 400 units of Superscript™ III Reverse Transcriptase (Life Technologies Corporation, Carlsbad, CA), 40 units of RNAseOUT (Life Technologies Corporation, Carlsbad, CA), PC2 reaction buffer (50 mM Tris–HCl pH 9.1, 16 mM ammonium sulfate, 3.5 mM magnesium chloride, and 150 μg/mL BSA) (AB Peptides, St. Louis, MO), 500 μM dNTPs (Clontech, Mountain View, CA), and 5 mM DTT (Life Technologies Corporation, Carlsbad, CA). The RNA was denatured at 65°C for 5 min, followed by ramping extension temperatures at 25°C for 10 min, 42°C for 60 min, 50°C for 30 min, 55°C for 30 min, and 70°C for 15 min.

### Amplification of HCV via nested PCR

The cDNA from the RT reaction was diluted 1:1 into a 40 μL PCR1 reaction mixture, containing PC2 reaction buffer (AB Peptides, St. Louis, MO), 200 μM dNTPs (Clontech, Mountain View, CA), 1.5 M betaine (Sigma Aldrich, St. Louis, MO), 2.56 units Klentaq DNA polymerase (AB Peptides, St. Louis, MO), 1.28 units *pfu* DNA polymerase (Stratagene, La Jolla, CA), and 400 μM each subtype-specific primer (Table 1). The PCR1 reaction was incubated at 94°C for 2 min, followed by 30 cycles at 94°C for 15 sec, 68°C −0.5°C/cycle (“touchdown” PCR) for 20 sec, followed by an incubation at 68°C for 12 min. The completed PCR1 reaction was diluted 1:10 into a second PCR mixture (PCR2, 50 μL). The reaction composition and PCR cycling parameters were identical to the PCR1 reaction, with the following changes; 3.2 units Klentaq DNA polymerase, 1.6 units of *Pfu* DNA polymerase, and nested subtype-specific primers were utilized (Table 1).

### Preparation of sample for sequencing

The PCR2 product (8991 bp) was analyzed and verified by agarose gel electrophoresis, and purified using the QIAquick 96 PCR Purification kit (QIAGEN, Valencia, CA). The purified PCR2 product was subsequently quantified using a NanoDrop 8000 Spectrophotometer (Thermo Scientific, Hudson, NH) prior to sequence analysis.

## Abbreviations

bp: Base pair; cDNA: Complementary DNA; DAA: Direct-acting antiviral; dNTP: Deoxyribonucleotide triphosphate; DTT: Dithiothreitol; LLOD: Lower limit of detection; HCC: Hepatocellular carcinoma; HCV: Hepatitis C virus; IU/mL: International units per milliliter; NS: Nonstructural; RBV: Ribavirin; RT-PCR: Reverse-transcriptase polymerase chain reaction; tRNA: Transfer RNA; UTR: Untranslated region.

## Competing interests

Authors are current or former employees of Vertex Pharmaceuticals Incorporated and may own stock or stock options in that company.

## Authors’ contributions

TLK, DJB, JDF, JL, SS, BS participated in the design, development and performance of the assay, as well as the sequence alignment and analyses. AK and CL helped with the design of the assay. YZ and EZZ participated in the optimization, modification, and finalization of the assay. EZZ, DJB, and TLK drafted the manuscript. All authors read and approved the final manuscript.

## Authors’ information

Benjamin Shames and Ann Kwong were employees of Vertex Pharmaceuticals Incorporated at time of study.
